# Mapping evidence of interventions and strategies to bridge the gap in the implementation of the prevention of mother-to-child transmission of HIV programme policy in sub-Saharan countries: A scoping review

**DOI:** 10.4102/phcfm.v9i1.1368

**Published:** 2017-05-29

**Authors:** Wilbroda H. Ngidi, Joanne R. Naidoo, Busisiwe P. Ncama, Zamasomi P. B. Luvuno, Tivani P. Mashamba-Thompson

**Affiliations:** 1 School of Nursing and Public Health, University of KwaZulu-Natal, South Africa; 2 Discipline of Public Health, University of KwaZulu-Natal, South Africa

## Abstract

**Background:**

Prevention of mother-to-child transmission (PMTCT) of HIV is a life-saving public health intervention. Sub-Saharan African (SSA) countries have made significant progress in the programme, but little is known about the strategies used by them to eliminate mother-to-child transmission of HIV.

**Aim:**

To map evidence of strategies and interventions employed by SSA in bridging the implementation gap in the rapidly changing PMTCT of HIV programme policy.

**Methods:**

Electronic search of the databases MEDLINE, PubMed and SABINET for articles published in English between 2001 and August 2016. Key words included ‘Sub-Saharan African countries’, ‘implementation strategies’, ‘interventions to bridge implementation gap’, ‘prevention of mother-to-child transmission of HIV’ and ‘closing implementation gap’.

**Results:**

Of a total of 743 articles, 25 articles that met the inclusion criteria were included in the study. Manual content analysis resulted in the identification of three categories of strategies: (1) health system (referral systems, integration of services, supportive leadership, systematic quality-improvement approaches that vigorously monitors programme performance); (2) health service delivery (task shifting, networking, shared platform for learning, local capacity building, supportive supervision); as well as (3) community-level strategies (community health workers, technology use – mHealth, family-centred approaches, male involvement, culturally appropriate interventions).

**Conclusion:**

There are strategies that exist in SSA countries. Future research should examine multifaceted scientific models to prioritise the highest impact and be evaluated for effectiveness and efficiency.

## Introduction

Sub-Saharan African (SSA) countries are highly burdened by HIV.^1^ The children are at risk of transmission of HIV from mother-to-child, which is a major public health concern. The programme ‘prevention of mother-to-child transmission (PMTCT) of HIV’ is aimed at addressing this problem. It has been demonstrated that the risk of mother-to-child transmission (MTCT) of HIV can be reduced by implementing key interventions, especially in resource limited settings. Countries are working towards^2,3^ meeting the Global Plan targets of the elimination of MTCT of HIV. Also, eradicating the HIV epidemic by 2020 remains the key target of the Sustainable Development Goal on health and well-being.^1,3,4,5^

Sub-Saharan African countries have demonstrated significant progress in the scaling up of PMTCT. The number of new HIV infections among children had significantly dropped from 350 000 in 2009 to less than 199 000 by 2013, which is a 43% decline in SSA countries. However, this is not in tune with the 90% reduction aimed for in the Global Plan (2011–2015).^4^ Disparities exist in the reduction of new HIV infection in children in the SSA countries. It is clear that the progress is slow, and efforts to accelerate progress are needed for countries to achieve the universal targets of 90-90-90.^1,3,4,5,6^

Much evidence exists demonstrating barriers to PMTCT programme policy implementation,^2,7,8,9,10,11,12,13^ meant to save lives. While World Health Organization (WHO) had recommendations for countries to adopt including Option B+ which promotes universal life-long antiretroviral treatment (ART) for all, crucial barriers to successful implementation remain.^4,9,14,15^ Strategies are employed by the SSA countries to bridge the implementation gap for the PMTCT programme. As appreciation of the impact of implementation of policy develops interest, it is crucial that the evidence of such interventions is identified and mapped out.

The aim of the scoping was to map the existing literature on the interventions and strategies applied in the SSA countries to close PMTCT of HIV policy implementation.

## Methods

### Search strategy

The scoping review involved a comprehensive systematic literature search in MEDLINE, PubMed, SABINET databases, including the reference list of included studies and key websites from WHO and UNAIDS. Two independent reviewers (WH and ZL) screened all titles and abstracts using predetermined inclusion and exclusion criteria, reviewed full text articles for inclusion, with disagreements settled by third and fourth reviewers (BN, JN) to ensure reliability of findings. The keyword search included ‘sub-Saharan African countries, implementation gap strategies, interventions to bridge implementation gap, prevention of mother-to-child transmission of HIV, closing implementation gap for PMTCT’.

The search was limited to English language publications between 2001 and August 2016. Studies were included if they reported on interventions or strategies for bridging implementation gap for PMTCT of HIV health programme policy in SSA countries; if they were fully accessible; and if they dealt with public health setting strategies or community-level interventions.

We excluded studies that were not published in English; not conducted in SSA countries, did not provide the setting; did not provide strategies; focused their intervention on programmes other than PMTCT; had inaccessible full texts; had inappropriate methodology; had comments or debates; were published prior to 2001.

## Data extraction and analysis

The search criteria used PRISMA 2009 diagram tool^16^ to map relevant searched articles (see Figure 2). A standardised data extraction form was used to guide eligibility of extracted studies edited from PICOS framework (Population, Intervention, Comparison, Outcome, Study design) which charted information on author and year, study design, population, % of population, intervention, aim of the study as well as the key findings including recommendations if any (see Table 1). The review was not limited to study designs. Manual content analysis was used to identify key categories and reporting on key findings. Mixed Method Appraisal Tool (MMAT) was used for quality assessment of included studies.^17,18^

**TABLE 1:** Summary of studies identified.

Author and year	Study setting	Study design	Population	% of the target population	Intervention	Main aim of the study	Results
Youngleson et al. 2010	South Africa, Cape Town, Cape Metro district	Operational research	Primary health care sites	All primary care sites in Cape Metro district	Health system intervention consisted of quality-improvement framework (system changes to identify and improve performance gaps; learning network to accelerate peer-to-peer learning approach that uses data to monitor improvement), policy and protocol changes as well as targeted resource addition	To reduce transmission of HIV from mother to infant, through health system strengthening approaches	PMTCT processes and outcome improvement included reduction in infants testing positive, from 7.6% to 5%. Spread of successful changes: postnatal testing improved from 79% to 95%. **Recommendation**: System improvement approach is needed to rapidly improve the performance of PMTCT implementation programme at scale in SSA; however, it requires clear design, leadership buy-in, local capacity building and reliable data system.
Ditekemena et al. 2012	SSA countries	Literature review	Studies that reported male involvement in MCH services	34 studies	Male participation in MCH and PMTCT services	To identify determinants of male partners’ involvement in MCH activities, focusing specifically on HIV PMTCT	Few interventions addressing male involvement and participation in MCH services. Identified three main determinants for male participation in PMTCT services: (1) socio demographic factors (level of education, income status), (2) health services–related factors (hours of health service operations, staff behaviour and space), (3) Sociologic factors (beliefs, attitudes). **Recommendation:** Improvement in PMTCT programme requires interventions addressing male involvement to create more male-friendly services and health education campaigns to changebeliefs and attitude.
Haines 2004	Low-income countries	Systematic reviews	Policymakers, public and health service providers	A total of 180 studies	Strategies to promote the uptake of research findings in low- and middle-income countries	To provide an overview of the effective approaches used to encourage the uptake of research findings for three main groups: policymakers, the public and health service providers	Strategies such as outreach from existing facilities, social marketing, supportive supervision and application of the principles of quality assurance can result in increased coverage of evidence-based interventions. **Recommendation:** To reckon that the effect of interventions varies per setting and behaviour that is targeted.
Whitworth 2010	SSA	Systematic review	SSA	Countries of SSA	Building research capacity in MCH in Africa	To understand issues of implementation in SSA countries in Maternal Newborn and Child Health (MNCH)	There is a need to broaden the base for health research in SSA countries, especially for implementation research. Addressing issues at implementation level. **Recommendation**: Strong health research systems that address bottlenecks to upscaling of effective interventions are needed; however, support is needed for African scientists, institutions and systems to solve African problems.
Ssengooba et al. 2011	Uganda	Case study: Pure qualitative study	Policymakers, technical officers, funders, researchers, print and media journalists	30 stakeholders (8 researchers, 12 policymakers, 10 media journalists)	A comparison of three domains of interest; lessons learnt from PMTCT and SMC, and evidence that drives policy	To understand the process of translating research into policy in order to improve health outcomes related to national health priorities in Uganda	Factors that facilitate PMTCT uptake and implementation included shared platform for learning, decision-making among stakeholders and implementation pilots to assess feasibility of intervention. **Recommendations**: Implementation research should be prioritised to guide policy processes. Feasibility should be determined of implementing new innovations on a large scale in different contexts of the research environment.
Turan and Nyblade 2013	Low--income settings	Review of literature	1599 articles	The final review included findings from 150 documents mainly from peer-reviewed journals	Conducted a strategic literature review and scan of both peer-reviewed and grey literature	To examine how HIV-related stigma affects utilisation of the series of steps that women must complete for successful PMTCT	Key steps and strategies include: (1) identifying, addressing and monitoring stigma in health services for pregnant and childbearing women; (2) listening to pregnant women living with HIV and designing programmes that directly address their needs; (3) involving women living with HIV in PMTCT service delivery; (4) reaching out to and positively engaging the communities and male partners of pregnant women living with HIV; (5) designing PMTCT media campaigns with the participation and input of advocacy groups and pregnant women living with HIV.
Doherty et al. 2009	South Africa one district in KwaZulu-Natal province, Amajuba	Operational research	PHC clinics, facility managers and lay counsellors	18 PHC clinics, with 15 facility manager interviews and 35 lay counsellors interviews	Participatory assessment phase followed by a feedback and planning phase and then an implementation and monitoring phase	To report on the results of a participatory intervention to improve an integrated PMTCT programme in a rural district in South Africa	Assessment highlighted weaknesses in training and supervision. Routine data use can reveal bottlenecks and poor coverage of programme indicators. Improvement in processes: CD4 testing improved from 40% to 97%; PCR testing from 24% to 68%; infant NVP from 15% to 68%; maternal NVP from 57% to 96%. **Recommendation:** Quality-improvement intervention using participatory data-driven approach can help improve programme performance.
Horwood et al. 2010	South Africa, Amajuba and UThukela districts, KwaZulu-Natal	A quantitative, cross-sectional descriptive study	Mothers in postnatal wards and immunisation clinics; antenatal and child health records reviews, nurses and lay counsellors in primary health care clinics	A total of 872 participants	An evaluation of routine implementation of the PMTCT programme, and the integration of PMTCT with routine maternal and child health services	To evaluate PMTCT implementation and integration of PMTCT with routine maternal and child health services	Programme performance improvement noted. About 47% of exposed babies had a PCR test, 91.3% received NVP, 33.1% had results for cd4 recorded, 47% received co-trimoxazole, a dedicated PMTCT nurse in 12 of 26 clinics. Roles not clear, leading to confusion of roles among health workers. **Recommendation:** Integration of PMTCT services into routine care; role clarification of health workers and record keeping are key interventions to prevent barriers to accessing services.
Kasenga et al. 2009	Malamulo SDA hospital in Thyolo district, Makwasa, Malawi	Retrospective record review	One hospital which has 15 mobile sites with two health centres	Three hospital-based registers were analysed from 2005 to 2007	Free maternity services were introduced at Malamulo hospital as an intervention to cater to the surrounding 15 villages	To study the implications of policy changes on the demand for antenatal care, HIV testing and hospital delivery among pregnant women in rural Malawi	Programmatic improvement: HIV testing increased from 52.6% to 98.8%, with 15.6% testing positive after free maternity services that is believed to have increased access to antenatal services. The programme reached 4528 pregnant women during the study period. 52.6% tested positive, delivered in hospital, and got NVP. **Recommendation:** Maternity services availability to clients, affordability of health services, involvement of service providers, communities and families, in designing and maintenance of service delivery is needed for programme uptake improvement. Economic constraints should be addressed.
Rollins et al. 2014	Malawi, Nigeria, and Zimbabwe	Qualitative participatory research	Policymakers, district health workers, academics, implementing partners and persons living with HIV	Between 42 and 70 representatives attended each workshop	Two-day workshops using the CHNRI process to identify barriers and formulate questions to address them	To describe the process used for prioritising PMTCT IR) questions	Health systems approaches for integrating and decentralising services or increasing access and uptake to interventions were consistently prioritised. Bridging the barrier between health facilities and communities and male involvement was ranked as important after health system. **Recommendation**: CHNRI promotes country ownership and helps direct strategic allocation or research resources.
Bhutta et al. 2005	Developing countries	Meta-analyses of RCTs	Review of available published and unpublished data	A total of 186 studies from developing countries were identified for in-depth review	The principal reviewers independently evaluated the data, and a common reporting matrix was used in summarising the findings. Studies were evaluated for size, setting, quality and design	To identify the impact of community-based strategies and interventions on perinatal and neonatal health status outcomes	Identifies a package of priority interventions to include in programmes and formulates research priorities for advancing the state of the art in neonatal health care. RCTs: 31 community-based RCTs reported primary neonatal health outcomes, and 40 reported secondary neonatal health outcomes. Only 10 studies were interventions conducted in health system settings or effectiveness trials. Most interventions had been tested on relatively small numbers of individuals. **Recommendations**: Importance of health system research, as well as evaluation of interventions to assess impact.
Kinney et al. 2010	SSA countries	Literature review	SSA countries	N/A	Review of literature on SSA countries to identify high impact opportunities for saving lives	To present known interventions to prevent child deaths and coverage of the interventions in SSA countries	Critical understanding of where and why the deaths occur, strategic data-based prioritisation is essential for progress. Scientifically proven interventions are available; however, they are underutilised to save the woman and child’s lives. **Recommendation**: Essential evidence-based interventions for MNCH, including PMTCT, can save many lives when it reaches everyone. A need to prioritise interventions using health service coverage data.
Friberg et al. 2010	SSA countries	Literature review	46 SSA countries	42 countries of SSA	Included all interventions in LiST (Lives saved tool). Doing modelling to determine lives saved	To estimate lives that could be saved by scaling up proven health interventions in health systems	Contexts count in selecting interventions. Outreach interventions/community-based interventions can save lives. Local data and differing health system settings are necessary. **Recommendation**: Outreach services and facility-based care is required for functioning health system. Use of local data to prioritise effective interventions is crucial.
Barker et al. 2015	South Africa	Descriptive study	Health facilities	South African Department of Health facilities	Quality-improvement methods to improve the performance of the PMTCT programme (model for improvement includes identification of an outcome goal based on best-available evidence, a set of measures to track progress towards that goal and a systematic way to test local ideas to close performance gaps)	To demonstrate how quality-improvement methods played a significant part in PMTCT improvements	The scale-up of the quality-improvement approach contributed to a dramatic fall in the nationally reported transmission rate for MTCT of HIV. By 2012, measured infection rate of HIV-exposed infants at around six weeks after birth was 2.6%, **Recommendation**: Quality-improvement methods can be used to improve reliability of complex treatment programmes delivered at the primary care level
Ezeanolue et al. 2016	Nigeria	Qualitative study	Policymakers, programme implementers, researchers	145 individuals (10 groups)	Engagement of stakeholders to identify interventions and implementation strategies to improve PMTCT	To advance research and practice related to PMTCT in Nigeria	25 unique interventions and implementation strategies including included (1) HIV diagnosis, (2) HIV diagnosis and linkage to care,(3) linkage and retention, as well as policy and data needs. **Recommendation**: Involvement of state, policymakers, Implementers and researchers help ensure relevancy.
Byamugisha et al. 2010	Uganda, eastern Uganda, Mbale district	Cross-sectional study – Qualitative	Men whose spouses were attending antenatal care at Mbale Regional Referral Hospital	388 men	Male involvement in spouses of women attending antenatal clinic	To determine the level of male involvement and identify its determinants in the PMTCT programme	The majority (74%) had a low male involvement index and only 5% of men accompanied their spouses to the antenatal clinic. **Recommendation:** Improvements in the health care system and community sensitisation of men about the benefits of antenatal care and the PMTCT programme are essential for mitigating the effect of socioeconomic and cultural factors.
Chabikuli et al. 2013	Nigeria	Records review	Public health care facilities	60 public health care facilities	Evaluation of service improvement intervention	To assess improvement, or lack thereof, in the uptake of PMTCT services at selected sites	About 120 537 women attended an ANC for the first time. Average of 167.4 monthly attendances per facility. ANC attendance increased per facility by 11.1 women monthly, (*p* < 0.01). The uptake of HIV testing was 87%, ARV prophylaxis uptake rose from 3.3 to 5.4 women per facility per month (*p* < 0.01). **Recommendation:** Service improvement intervention improved the utilisation of PMTCT services.
Kalembo and Zgambo 2012	SSA countries	Literature review	678 articles	44 articles	Searched peer-reviewed public research articles in SSA countries	To explore how LTFU has affected the successful implementation of PMTCT programme in SSA countries	Health facility factors, stigma and discrimination, and socioeconomic factors contribute to LTFU. **Recommendation**: Strategies to address LTFU includes psychosocial support, family-focused approach and monitoring and evaluation of health information systems.
Wettstein et al. 2012	SSA countries	Systematic review and meta-analysis	PMTCT studies in SSA countries	44 studies from 15 countries	Systematic review to identify missed opportunities to PMTCT	To determine reasons of loss to programme and poor PMTCT ART coverage in SSA countries	Programme performance improvement: HIV testing uptake increased to 94%, ART coverage improved to 70%, 64% infants tested for HIV to determine transmission. Improved PMTCT interventions noted when male partner is involved or if treatment was provided in facility. **Recommendation**: An integrated family-centred approach improves retention.
Woldesenbet et al. 2015	South Africa, 9 provinces	Cross-sectional survey – Quantitative study	Mother and care-giver in public health facilities	580 public health facilities (10 820)	To estimate the population attributable fraction associated with dropouts at each service point	To measure national uptake of antenatal and early postnatal PMTCT services, and identify key dropout points	High dropout rate, especially among adolescent mothers (34.9%). Of 31.7% mothers tested positive, 80.4% received ART. 85.2% of adolescent mothers were unaware of their status. **Recommendation**: Community-level interventions and health facility–level interventions are required to identify the risk factors for missed opportunities for PMTCT, including raising community awareness.
Mate et al. 2013	South Africa, 5 districts	Operational research	NGO partners, health facilities	6 NGOs, 5 districts (181 facilities)	Improving DOH–NGO collaboration using Quality-Improvement model involving: setting targets, improving data, simplifying processes and building networks	To reduce MTCT rates to <5% in all intervention districts through engaging partners	Accelerated plan used to improve PMTCT services at health care facilities. A total of 676 health care workers and managers were trained in quality-improvement methods and tools. Coverage of seven key processes in the PMTCT programme was tracked on a monthly basis. **Recommendation**: Network models could successfully recruit key stakeholders into strong partnerships, leading to rapid scale-up of public life-saving interventions.
Ibeto et al. 2014	South Africa, Cape Town, Khayelitsha	Case series record review	Health facility records	Community health centre records	Investigation of each HIV-positive PCR baby records review	To establish possible causes of transmission of HIV-infected infants to identify obstacles to PMTCT	Reduction of PCR-positive infants to 1.6%. A total of 926/1158 (80%) of exposed infants had PCR results, with 15/926 (1.6%) PCR-positive. Main risk factors for elimination of mother-to-child transmission (EMTCT): late presentation for antenatal care, inadequate PMTCT prophylaxis and lack of viral load monitoring. **Recommendation**: PMTCT programme must consider each PCR-positive infant to provide with insight into correcting clinical and programmatic reasons for HIV transmission.
Herce et al. 2015	Malawi, 5 districts	Cross-sectional study	Government health facilities	5 districts’ health facilities	Health worker training and mentorship; HIV counselling for couples and testing; male partner involvement; psychosocial support for women	To evaluate performance on PMTCT indicators	Programme performance of facility-level outcome indicators improved. HIV counselling and testing improved from 66% to 87%, ART coverage from 23% to 96%, NVP infants from 1% to 100%, PCR and testing from 52% to 62%. **Recommendation**: Continued investments are needed to strengthen PMTCT cascades.
Mahmud et al. 2010	Malawi, rural hospital	Evaluation study –Quantitative	Community care workers in rural setting in Malawi	75 community care workers in rural setting in Malawi	Text message–based interventions using mHealth by community care workers (trained on its utilisation)	To use mHealth interventions in bridging the patients–physicians gaps	Reduced operational costs; saved on worker time; fuel saving. **Recommendations**: mHealth interventions can provide cost-effective solutions to communication barriers in rural settings.
Leon et al. 2013	South Africa, Cape Town	Qualitative study – Longitudinal study	Staff and patients in health care setting	4 primary health care services’ nursing staff, lay counsellors, leadership team, facility managers, trainers, patients	PICT	Process evaluation of PICT intervention	PICT embedded in practice leads to improvement in counselling and testing. **Recommendation**: Strong senior leadership; implementation support; accountability mechanism; responsive organisational context.

*Source*: Adapted from Arksey and O’Malley^47^

ANC, antenatal clinic; ART, anti-retroviral treatment; CHNRI, Child Health Nutrition Research Initiative; LFTU, lost to follow up; IR, implementation research; MCH, maternal and child health; mHealth, mobile health; NVP, nevirapine; PCR, polymerase chain reaction; PICT, provider-initiated counselling and testing; PMTCT, prevention of mother-to-child transmission of HIV; RTCs, randomised controlled trials SSA, sub-Saharan Africa.

### Ethical consideration

This article is part of a PhD thesis, which is being currently conducted in accordance with permission from the ethics committee of the University of KwaZulu-Natal (UKZN) BREC (Biomedical Research Ethics Council), under the protocol reference number BE112/14.

## Review results

The three-step study selection process for this scoping review comprised (1) first step: database search using keywords and title screening that returned 734 articles; 11 additional records were identified through reference lists and websites for WHO and UNAIDS. A total of 329 records after exclusion criteria, duplicates and non-English records were excluded; (2) second step: screening of titles and abstracts identified 73 records for full text review after exclusion of 256 papers which were either not conducted in SSA countries or reported on strategies or interventions for other health programmes and not for the PMTCT programme; (3) third step was full article screening, done in duplicate on 73 records, which resulted in 44 records being excluded for providing insufficient information; no description of interventions or strategies of interest; the full article not being accessible; for being in comment or debate form of report and conducted prior to 2001. This resulted in 29 records assessed for quality using MMAT tool checklist; however, a total of four articles were excluded for reasons such as inappropriate methodology or for not providing key findings (report on a protocol for randomised control trial). Average time spent critically appraising one article was 15 minutes. At the end of the final review and assessment, 25 eligible studies were included for final analysis. Details related to the search are provided in the PRISMA flow diagram (see Table 1).^19,20,21^

Papers were mainly from six specific countries as well as from studies done across SSA countries as follows: South Africa (*n* = 8); Nigeria (*n* = 2); Uganda (*n* = 2); Malawi (*n* = 3); and a study done in three different countries of Malawi, Nigeria and Zimbabwe (*n* = 1), including studies done in SSA countries (*n* = 9) not specifically in one country. Majority of studies conducted in SSA countries were systematic reviews (*n* = 9), studies reporting on operational research (*n* = 3), qualitative studies (*n* = 6), quantitative studies (*n* = 4) and record review studies (*n* = 3). There were neither randomised controlled trial studies nor studies of mixed methods.

### Types of strategies

Studies reported on the various stages of the implementation of PMTCT of HIV programmes by SSA countries ranging from the use of Option B to that of Option B+ as recommended by WHO.^3,6^

**FIGURE 1:**
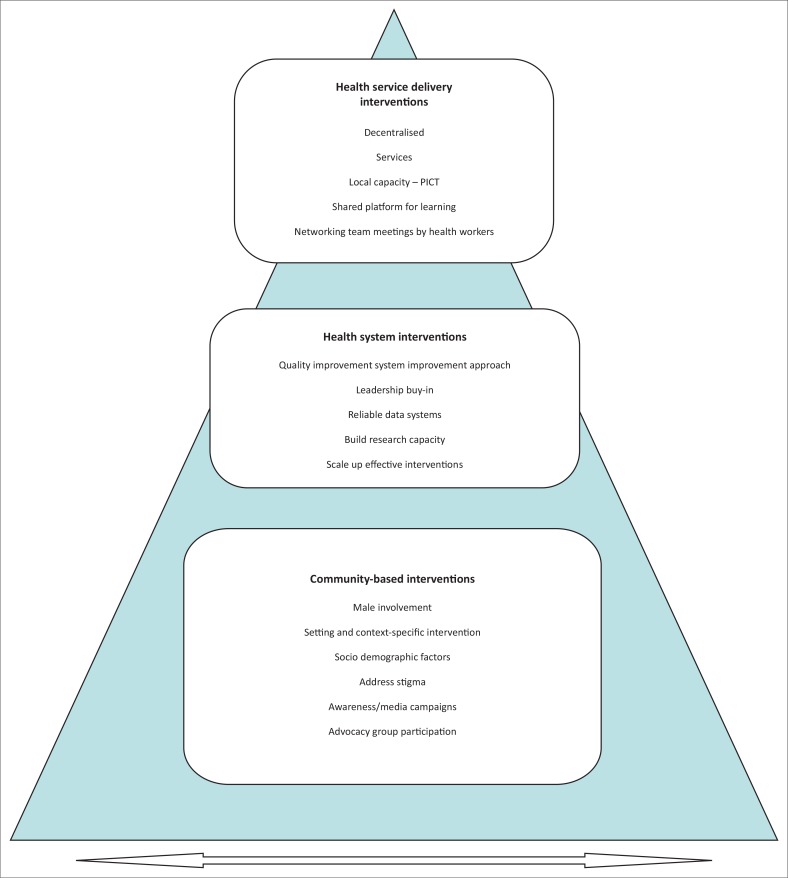
Model developed providing summary of three key categories from final articles.

After content analysis, the three most significant strategies and interventions that were in line with our objectives and inclusion criteria were categorised as follows: health service delivery, community-based interventions and health system strategies (see Figure 1).

#### Health service delivery strategies

The common strategies reported included shared platform for learning and decision-making among stakeholders,^22^ health service improvement of processes^2,23,24^ and data-driven quality-improvement approach which was mainly reported in South Africa.^25,26,27,28^ This approach included health workers forming information teams to discuss issues relating to performance, forming learning networks which also promote shared learning, and discussing changes and planning for implementation.

There were studies that reported on strengthening of integration of PMTCT services into maternal health programme as well as clear role clarifications among the implementers^8,10,29,30^; reinforcement of provider-initiated counselling and testing services by health workers to improve uptake^31^ which is also linked to task shifting. Another approach for assisting in bridging the gap of PMTCT implementation is monitoring individual infants to determine the reason for transmission so that interventions can be targeted at the barriers.^32^ This, according to the study, can reveal specific barriers to be addressed for countries to get to zero infections.

**FIGURE 2:**
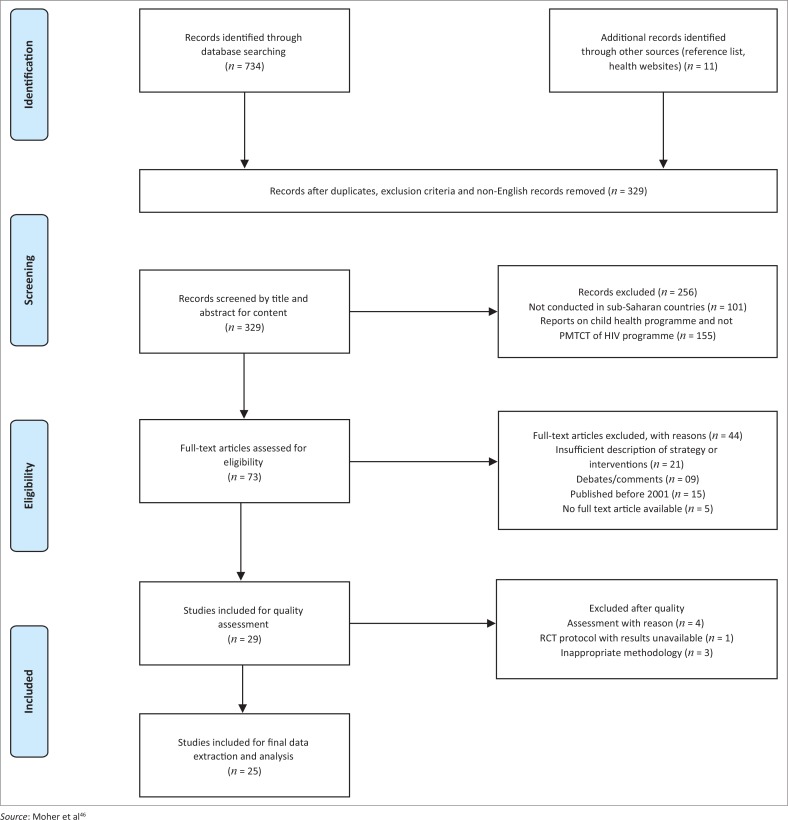
PRISMA 2009 flow diagram of searched articles.

#### Community-based strategies

It was common among assessed articles that the consideration of context and settings remained crucial, especially in SSA countries.^11,12,33,34^ The reality on the ground is seen as one of the major barriers to implementation of prevention programmes such as the PMTCT programme; hence, strategies should be tailor-made to cater to such barriers. The importance of community involvement in bridging the gap in policies remains crucial.^21,35,36,37^

There were articles that considered the promotion of disclosure of HIV infection in ensuring that communities are supported in the programme.^12^ According to health care workers, failure to disclose can result in poor support. Owing to the fact that the programme needs communities to take part for its success, linkage between public health facilities and communities was seen as one strategy to close gaps in implementation.

Some models included the community care-givers, strengthened the linkages and the referral system between communities and health facilities^37,38,39^ as well as made communities aware through community sensitisation models, including conducting campaigns in communities to create awareness and to address stigma which is reported as a barrier in communities.^34,37,40^

In the inclusion of families using family-centred approach^41^, male involvement^36,38^ was identified as a gap where only women seem to be the focus. This calls for more engagement of men in future studies. For some studies, innovations, including use of technology, for example use of text messages by community care workers (mHealth) to reach communities,^39^ demonstrate how technology can be utilised to access health.

Access to services remains crucial for any prevention programme to be a success; therefore, free and affordable services that can improve uptake of services are employed^41^; however, considering contexts, the affordability of such interventions across countries and sustainability of such an approach need to be further explored.

#### Health system interventions

Health system–related interventions included among others the importance of leaders in the health system to pave the direction for implementers; hence, leadership buy-in^27^ to effect change in the use of quality-improvement model for improvement is proposed for the implementation to take place. However, models on leadership engagement need to be explored for countries to maximise benefits from it.

The reviews highlighted the importance of using reliable data systems with the health workers as implementers and adopting an approach that addresses health system improvement^25,26,27,28^ as one of the strategies, for example the use of quality-improvement approaches towards improvement that promotes health system strengthening. WHO, in its framework of six building blocks, highlighted information as one of the health system–building blocks and the importance of monitoring health system performance of programmes for progress as well as impact.^23,42^

Strengthening research capacity among SSA countries is seen as vital in improving implementation. This included capacity building on implementation science research targeting health workforce, especially in resource limited countries.^4,27,43,44,45^ By doing so, it is believed that low-resource countries can better understand implementation science as it relates to answering critical research questions in their context.

Figure 1 represents the model to summarise identified key categories.

## Discussion

Our review on mapping strategies and interventions to bridge the gap in the implementation of PMTCT of HIV in the SSA countries had found articles on strategies and promising approaches used while identifying areas for future research. Based on this review, majority of papers are secondary papers (*n* = 9), reporting on systematic reviews and lack primary researches reported on this area. Papers also reported on barriers to implementation and enablers thereof with limited studies reporting on strategies for closing the implementation gap. It was common that for a strategy to be effective, it needed to be informed by identified barriers to implementation.^34^

Evidence regarding particular strategies mostly relates to the PMTCT programme performance through data indicators monitoring of the PMTC cascade. Previous evidence had mainly focused on monitoring performance for PMTCT indicators through the PMTCT cascade. This was used to measure programme implementation, and identified gaps in the PMTCT programme coverage which identified strategies that included using quality-improvement approaches in public health care settings that focus on obtaining and building will from leadership, ideas for changes, monitoring data as well as using platforms for shared learning which was common across studies.^2,25,26,27,32,48^

While task shifting emerged as one of the key solutions to bridge policy implementation gaps, and was seen as a strategy to improve healthcare coverage as well as address human resources challenges which were common across the SSA countries, inadequate supervision and poor role clarification remain a challenge.^8,49^

Majority of papers report on health worker interventions including health system intervention,^22,25,26,27^ with the majority reporting on the importance of engaging implementers for any implementation gap to be closed^26,27,31,39,43,44^ which was found to be crucial, especially in low-resource countries.

While the strategies were offered by the SSA countries, they lack evaluations to determine the effectiveness of those identified strategies.^44^ The lack of evaluation of implementation strategies hinders their assessment for effectiveness. Interventions reported including free service delivery^41^ may require further assessment for sustainability of such an approach in a given low-resource country; however, the majority of SSA countries were offering free maternity services.

With countries expected to eliminate MTCT of HIV, demand for countries to speed up implementation and package interventions and strategies that are effective in closing the implementation gap remain crucial.

### Strengths and limitations of this study

The review was limited to studies published in English, as it is the commonly used language for communication in the majority of SSA countries. The review was limited to articles published from 2001 to August 2016, and publications prior to this year were excluded for relevancy of the research topic as well as for reasons related to PMTCT not fully being implemented in SSA countries before 2001. The review focused on articles published in SSA countries, due to comparable settings and similar resources available.

Our review focused on the strategies and interventions for PMTCT of HIV programme to close the gap in implementation, and therefore excluded the broader aspect of other programmes that may offer strategies to close the gap in implementation; however, this review can guide future research so that a broader evidence of effective strategies used in health and other prevention programmes can be mapped.

## Conclusions and recommendations

Prevention of MTCT of HIV remains a growing global public health priority, more so in the highly HIV-burdened SSA countries. This review revealed that there is no one size fits all approach in closing the implementation gap for priority prevention programmes such as PMTCT of HIV in countries with low resources. There is a need for a combination of multifaceted approaches that consider contextual settings, leadership involvement, shared learning forums while considering resources towards closing the implementation gap.

Packaging of strategies that have proven to work for scale-up to similar settings is needed; however, evaluation of such interventions and strategies for effectiveness and efficiency is of vital importance. The Global Plan vision (2011–2015), of no child shall be born with HIV and keeping mothers alive as well as eliminating MTCT of HIV until it does not constitute a public health concern, as well as achievement of universal 90-90-90 strategies require unusual business approaches as well as the engagement with researchers, policymakers, implementers and communities to prioritise implementation in the policy development phase, considering various contextual settings.
